# COVID-19 Vaccine in Lung and Liver Transplant Recipients Exceeds Expectations: An Italian Real-Life Experience on Immunogenicity and Clinical Efficacy of BNT162b2 Vaccine

**DOI:** 10.3389/ti.2024.12729

**Published:** 2024-07-10

**Authors:** Letizia Corinna Morlacchi, Gianfranco Alicandro, Sara Uceda Renteria, Nunzio Zignani, Giovanni Giacomel, Valeria Rossetti, Michele Sagasta, Gaia Citterio, Andrea Lombardi, Clara Dibenedetto, Barbara Antonelli, Lorenzo Rosso, Pietro Lampertico, Ferruccio Ceriotti, Francesco Blasi, Maria Francesca Donato

**Affiliations:** ^1^ Respiratory Unit and Adult Cystic, Fibrosis Centre, Internal Medicine Department, Fondazione IRCCS Ca’ Granda Ospedale Maggiore Policlinico di Milano, Milano, Italy; ^2^ Department of Pathophysiology and Transplantation, Università degli Studi di Milano, Milano, Italy; ^3^ Department of Pediatrics, Cystic Fibrosis Centre, Fondazione IRCCS Ca’ Granda Ospedale Maggiore Policlinico, Milan, Italy; ^4^ Division of Clinical Pathology, Fondazione IRCCS Ca’ Granda Ospedale Maggiore Policlinico di Milano, Milano, Italy; ^5^ Division of Gastroenterology and Hepatology, Fondazione IRCCS Ca’ Granda Ospedale Maggiore Policlinico di Milano, Milano, Italy; ^6^ General Surgery—Liver Transplant Unit, Fondazione IRCCS Ca’ Granda Ospedale Maggiore Policlinico, Milano, Italy; ^7^ Thoracic Surgery and Lung Transplant Unit, Fondazione IRCCS Ca’ Granda Ospedale Maggiore Policlinico di Milano, Milano, Italy

**Keywords:** COVID-19 vaccination, solid organ transplant recipient, vaccine immunogenicity, lung transplant recipients, liver transplant recipients, humoral response, cell mediated response

## Abstract

This study assessed humoral and T cell-mediated immune responses to the BNT162b2 vaccine in orthotopic liver transplant (OLT) and lung transplant (LUT) recipients who received three doses of the vaccine from March 2021 at our institution. Serum samples were collected 60 days post-second and third dose to quantify antibodies against the spike region of SARS-CoV-2 while whole blood samples were collected to analyze the SARS-CoV-2-specific T-cell response using an IFN-γ ELISpot assay. We enrolled 244 OLT and 120 LUT recipients. The third dose increased antibody titres in OLT recipients (from a median value of 131 after the second dose to 5523 IU/mL, *p* < 0.001) and LUT recipients (from 14.8 to 1729 IU/mL, *p* < 0.001). T-cell response also increased in OLT recipients (from 8.5 to 23 IFN-γ SFU per 250,000 PBMC, *p* < 0.001) and LUT recipients (from 8 to 15 IFN-γ SFU per 250,000 PBMC, *p* < 0.001). A total of 128 breakthrough infections were observed: two (0.8%) OLT recipients were hospitalized due to COVID-19 and one died (0.4%); among LUT recipients, seven were hospitalized (5.8%) and two patients died (1.7%). In conclusion, the three-dose schedule of the BNT162b2 vaccine elicited both humoral and T cell-mediated responses in solid organ transplant recipients. The risk of severe COVID-19 post-vaccination was low in this population.

## Graphical Abstract

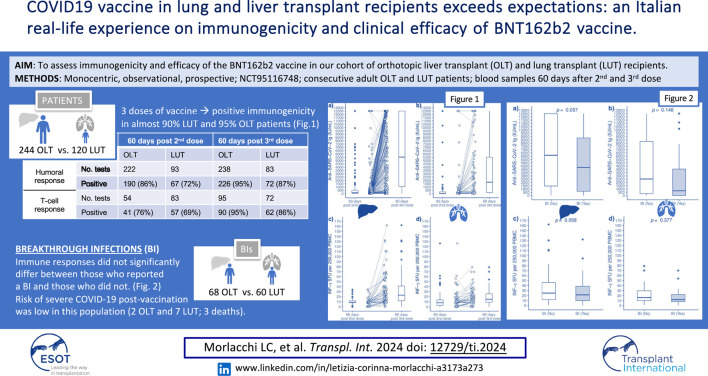

## Introduction

At present, no definitive data are available on the efficacy and the immunogenicity of anti-SARS-CoV-2 vaccines in solid organ transplant (SOT) recipients. The available evidence is inconsistent, and there is limited data on different type of SOT recipients and their cell mediated immune responses [1–4]. It is still unknown whether a specific level of serum antibodies may confer protection from infection or severe disease, and routinely measurements are not currently recommended. Conversely, mRNA vaccines appear to be safe in the transplanted population, and, at present, there are no concerns about the possible onset of rejection or other serious adverse events following their administration [5].

### Aim

The primary aim of this study was to assess the immunogenicity of the BNT162b2 vaccine in our cohort of orthotopic liver transplant (OLT) and lung transplant (LUT) recipients. As a secondary aim, we evaluated the occurrence and the severity of breakthrough infections (BI) in this population after the completion of a three-dose vaccination course.

## Patients and Methods

### Participants

We conducted a prospective, observational study enrolling consecutive OLT and LUT recipients attending our hospital. The study period extended from 1st March 2021, to 31st October 2022. We screened all patients living in Lombardy who received the vaccine at our hospital vaccination hub.

Patients were considered eligible for the vaccination if: time from transplantation was more than 3 months for OLT and 6 months for LUT; they had not recently received intensive treatment for rejection or any other clinical reason to wait for administering vaccinations; they had no history of allergy for any of the vaccine excipients. Patients were excluded if: they were under 18 years old; had COVID-19 between the administration of the second and the third dose of vaccination; lived outside Lombardy or refused to provide consent.

All patients received a three-dose schedule of the BNT162b2 vaccine (second dose was given 21 days after the first one; third dose was given 180 ± 30 days after the second dose); each dose was administered by intramuscular injection into the deltoid muscle.

Blood samples were collected 60 days after the second dose and again 60 days after receiving the third dose, in order to determine their anti-SARS-CoV-2 total Ig antibodies and T cell-mediated immune responses to vaccine. We were not able to perform T-cell mediated response analysis on the entire population due to high costs, complexity of the method and laboratory overloading activities at that time. Overall, 304 samples were analyzed for T-cell response. The first selection criterion was sampling time (specimens collected until the number of tests/kits available for analysis was exhausted, 10 kits after the second dose and 10 kits after receiving the third dose). The second selection criterion was to discard samples with <6 mL of whole blood collected and consequently with an insufficient peripheral blood mononuclear cells PBMCs for analysis.

The following data were collected: date of birth and transplantation, body mass index (BMI) at enrollment, etiology and indication for transplantation, prior COVID-19, post-transplant comorbidities (including diabetes, chronic kidney disease and cancer), immunosuppression regimen and graft function. The latter was evaluated with liver stiffness (measured by Fibroscan^®^) for OLT recipients and with pulmonary function tests criteria for chronic lung allograft dysfunction (CLAD) for LUT patients, as defined in the ISHLT 2019 consensus document [6].

All patients received traditional follow up, with regular visits in our outpatient clinics (every two-three months for LUT recipients and every four-six months for OLT recipients); patients were also instructed to contact our center by means of email and/or phone call in case sentinel symptoms occurred in order to give them appropriate indication. No patient was lost to follow up.

The study was registered on Clinicaltrials.gov (NCT 05116748, COVID-19_VaxSOT).

### Endpoints

The primary endpoints of the study included the anti-SARS-CoV-2 antibody titre and Interferon-γ (INF- γ)- secreting T cells measured 60 days after the second and third dose of the vaccine.

The laboratory procedures used for the quantification of humoral and T cell-mediated immune responses are reported in the Supplementary Material.

The secondary endpoints were the incidence of BI, including both asymptomatic/paucisymptomatic and severe forms. Additionally, the study collected unusual adverse events as well as serious adverse events (SAEs) and sentinel events.

### Definitions of COVID-19 Outcomes

A BI was defined as an infection occurring 14 days or more after receiving the third dose [7], and it was documented by an RT-PCR test or antigenic test.

Severe COVID-19 was defined as SARS-COV2 infection requiring hospitalization and/or causing pneumonia, respiratory failure, sepsis, septic shock, acute respiratory distress syndrome or death.

COVID-19 related mortality was defined as a death with COVID-19 listed in the death certificate.

### Statistical Analysis

Before the analysis, any antibody and IFN-γ SFU values that fell below the lower limit of quantification (LLOQ) were replaced with a value equal to 0.5 times the LLOQ. If any values exceeded the upper limit of quantification (ULOQ) and the actual values were not available, they were substituted with the ULOQ.

The data were presented as median and interquartile range (25th–75th percentile). Linear quantile mixed models with subject-specific random intercept were used to identify potential predictors of humoral and T cell-mediated responses [8]. We chose these models over other regression models because they do not assume a normal distribution of the response variable and are less sensitive to outliers. Separate models were fitted for OLT and LUT recipients, with anti-SARS-CoV-2 antibodies or INF-γ SFU per 250,000 PBMC as the response variables, and sex, age at vaccination, time from transplantation, prior SARS-CoV-2 infection, comorbidities potentially affecting immune responses, immunosuppressive therapy and vaccine dose (post third dose *vs* post second dose) as predictors.

Rates of BI were computed by dividing the number of BI by person-days and then multiplied by 1,000. Person-days were computed from 14 days after receiving the third dose until 31 October 2022 (the end of the study). The infection rate ratio (IRR) was computed with the rate observed among LUT recipients in the numerator and that observed among OLT recipients in the denominator, with the 95% confidence interval (95% CI) obtained using the Poisson distribution. Humoral and cell mediated responses measured after the administration of the third dose were compared between patients who reported a BI and those who did not within each SOT group using the Wilcoxon sum rank test. All statistical tests were two-sided and *p*-value<0.05 were considered statistically significant.

### Ethics

This study received approval from the ethics committee of the I.R.C.C.S. Istituto Nazionale per le Malattie Infettive Lazzaro Spallanzani (Parere n. 422 del Registro delle Sperimentazioni 2020/2021). Written informed consent was obtained from all subjects.

## Results

This study included 244 OLT recipients and 120 LUT recipients, whose characteristics are summarized in Table 1.

**TABLE 1:** Patient characteristics.

Characteristic	OLT recipients, *N* = 244^a^	LUT recipients, *N* = 120^a^
Sex		
Females	70 (28.7%)	52 (43.3%)
Males	174 (71.3%)	68 (56.7%)
Age at vaccination (years)	66.5 (58.5, 71.3)	43.0 (34.8, 57.0)
Age at transplantation (years)	56.2 (48.5, 62.5)	36.0 (27.0, 51.3)
Time from transplantation (years)	8.3 (3.4, 15.9)	5.0 (4.0, 8.0)
BMI (kg/m^2^)	25.0 (23.0, 27.0)	22.4 (20.5, 25.7)
BMI category		
Normal weight	98 (40.2%)	72 (60.0%)
Underweight	3 (1.2%)	11 (9.2%)
Overweight	103 (42.2%)	30 (25.0%)
Obesity	40 (16.4%)	7 (5.8%)
Indication for transplantation		
Decompensated cirrhosis	123 (50.4%)	—
Hepatocellular carcinoma	116 (47.5%)	—
Fulminant hepatic failure	5 (2.0%)	—
Cystic fibrosis	—	75 (62.5%)
COPD	—	10 (8.3%)
Idiopathic pulmonary fibrosis	—	14 (11.7%)
Other pulmonary diseases	—	21 (17.5%)
Cardiovascular disease	37 (15.2%)	23 (19.2%)
Diabetes	82 (33.6%)	94 (78.3%)
Cancer (excluding non-melanoma skin cancer)	42 (17%)	10 (8.3%)
Non-melanoma skin cancer	9 (3.7%)	16 (13.3%)
Chronic renal failure	54 (22.1%)	65 (54.2%)
Graft function		
FibroScan ≥8 kPa^b^	52 (21.6%)	—
Chronic lung allograft dysfunction	—	31 (25.8%)
Previous SARS-CoV-2 infection	12 (4.9%)	12 (10.0%)
Immunosuppression regimen		
Prednisone	33 (13.5%)	120 (100.0%)
Mycophenolate mofetil/Azathioprine	168 (68.9%)	55 (45.8%)
Tacrolimus/Cyclosporine	244 (100.0%)	120 (100.0%)
Triple immunosuppression	19 (7.8%)	91 (75.8%)

BMI, Body mass index; COPD, Chronic obstructive pulmonary disease; OLT, Orthotopic liver transplant; LUT, Lung transplant.

^a^ n (%); Median (IQR).

^b^ Data not available in three patients.

A total of 636 measurements of anti-SARS-CoV-2 total Ig antibodies and 304 measurements of T-cell responses were obtained. Their distribution by group (OLT vs. LUT recipients) and time (60 days after the second dose vs. 60 days after the third dose of the vaccine) is presented in Table 2, along with the percentage of measurements above the positive response threshold.

**TABLE 2:** Number of measurements and positive humoral and T cell-mediated responses by organ transplant group (liver of lung) and time from COVID-19 vaccine doses.

Transplanted organ	Time	No. of antibody measurements	No. of T cell- mediated response measurements	Positive antibody responses (% of total measurements)	Positive T cell-mediated responses (% of total measurements)
Liver	60 days post 2nd dose	222	54	190 (85.6)	41 (75.9)
	60 days post 3rd dose	238	95	226 (95.0)	90 (94.7)
Lung	60 days post 2nd dose	93	83	67 (72.0)	57 (68.7)
	60 days post 3rd dose	83	72	72 (86.7)	62 (86.1)

**FIGURE 1:**
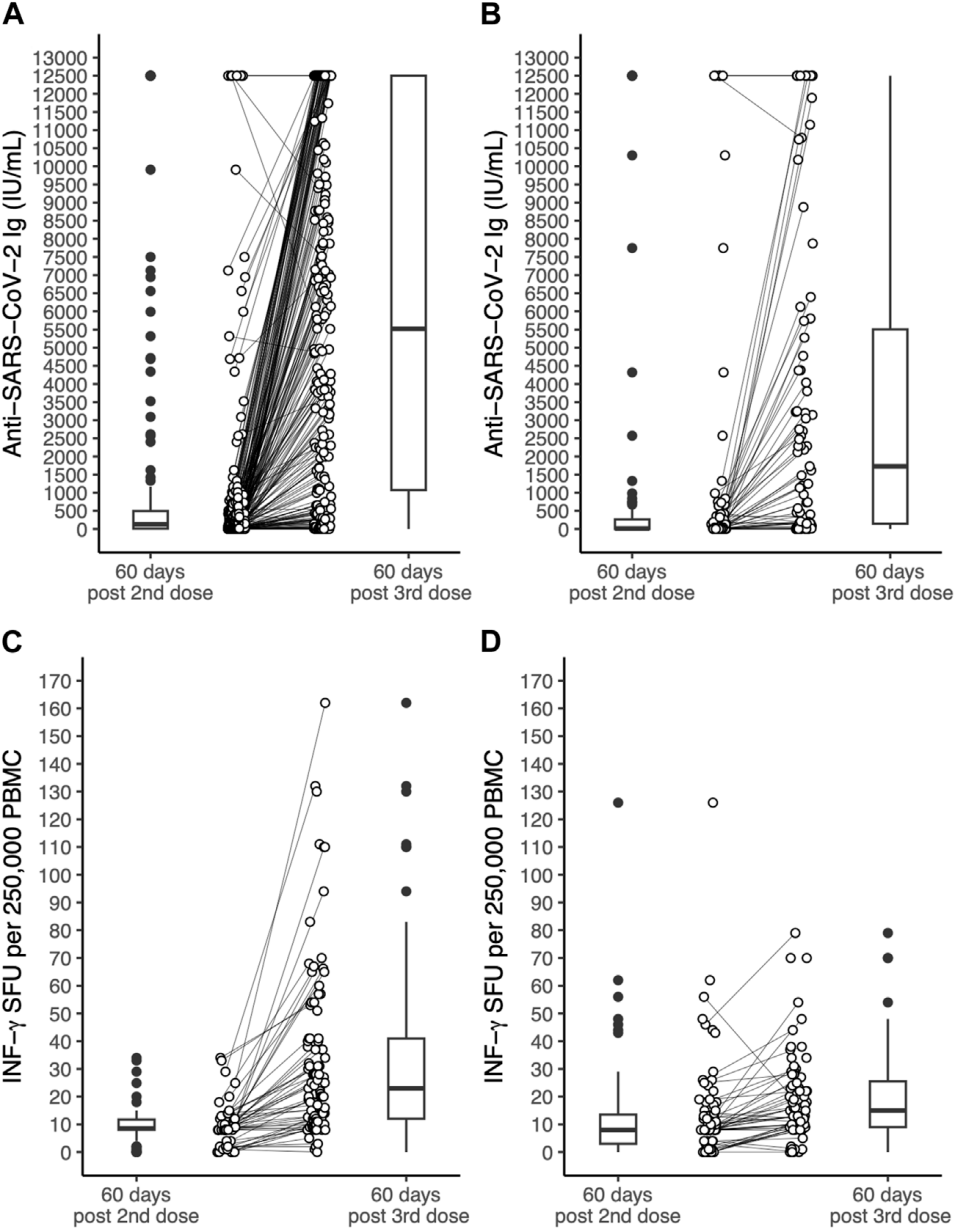
Serum concentration of antibodies to the SARS-CoV-2 spike protein receptor binding domain and INF-γ SFU measured in OLT recipients and LUT recipients 60 days after the 2nd dose of the BNT162b2 vaccine and 60 days after the 3rd dose. **(A)**: anti-SARS-coV-2 antibodies in OLT recipients. **(B)**: anti-SARS-coV-2 antibodies in LUT recipients. **(C)**: INF-γ SFU in OLT recipients. **(D)**: INF-γ SFU in LUT recipients. The lines within the boxes indicate the median, the edges of the boxes are the lower and the upper quartiles (the interquartile range), the lines extending from the box (whiskers) indicate the adjacent values (the most extreme values that are still within a distance of 1.5 times the interquartile range from the nearest quartile) and the black dots beyond the whiskers are outliers. The white dots indicate individual values and the lines join the measurement on the same subject after the 2nd and the 3rd dose. INF, Interferon; LUT, Lung transplant; OLT, Orthotopic liver transplant OLT; PBMC, Peripheral blood mononuclear cells; SFU, Spot forming units.

Serum concentration of anti-SARS-CoV-2 antibodies and IFN-γ SFU measured at 60 days after the second or the third dose in OLT and LUT recipients are shown in Figure 1.

Prior SARS-CoV-2 infection was significantly associated with higher antibody titres in both OLT and LUT recipients and with higher INF-γ SFU among LUT recipients. Among OLT recipients, chronic renal failure was associated with lower INF-γ SFU (expected difference in median: −11.2 INF-γ SFU per 250,000 PBMC). Age was associated with lower INF-γ SFU only among LUT recipients, with an estimated difference in median INF-γ SFU of −0.2 per 1-year increment. The third dose was associated with higher immune responses in both OLT and LUT recipients. The expected median increase in antibody titre after the third dose was 4976 IU/mL among OLT recipients and 2345 IU/mL among LUT recipients. The expected median increase in INF-γ was 21.2 SFU per 250,000 PBMC among OLT recipients and 5.8 SFU per 250,000 PBMC among LUT recipients. No significant associations were found for the remaining predictors considered in the models (Table 3).

**TABLE 3:** Results of the quantile mixed models aiming at identifying predictors of antibody and T cell-mediated responses to BNT162b2 vaccine against SARS-CoV-2 in liver and lung transplant recipients.

Response variable	Predictors	OLT recipients		LUT recipients	
		β (95% CI)	*p*-value	β (95% CI)	*p*-value
Anti-SARS-CoV-2 Ig (IU/mL)	Intercept	564 (−589; 1,717)	0.330	1,372 (−924; 3,669)	0.236
	Male sex	152 (−670; 975)	0.711	222 (−817; 1,261)	0.670
	Age (years)^a^	−4 (−34; 27)	0.799	−13 (−42; 16)	0.364
	Time from transplantation	8 (−25; 42)	0.630	−6 (−191; 179)	0.951
	Prior SARS-CoV-2 infection	7,351 (5,704; 8,997)	<0.0001	6,874 (3,668; 10,080)	<0.0001
	Diabetes	−155 (−970; 659)	0.704	−674 (−2,248; 900)	0.394
	Chronic renal failure	−678 (−1,531; 174)	0.116	−917 (−2,091; 258)	0.123
	Cancer	301 (−562; 1,164)	0.487	1,129 (−1,476; 3,734)	0.388
	Mycophenolate Mofetil/Azathioprine	−562 (−1,403; 279)	0.186	975 (−27; 1,977)	0.056
	Triple immunosuppression	−645 (−2,302; 1,012)	0.438	−960 (−2,560; 641)	0.234
	Vaccine dose (post 3rd vs. 2nd dose)	4,976 (4,354; 5,597)	<0.0001	2,345 (1,380; 3,309)	<0.0001
INF-γ SFU (per 250,000 PBMC)	Intercept	7.4 (−3.8; 18.5)	0.192	7.6 (−7.9; 23.1)	0.331
	Male sex	2.9 (−7.6; 13.4)	0.580	2.6 (−2.8; 8)	0.333
	Age (years)^a^	−0.1 (−0.4; 0.2)	0.492	−0.2 (−0.4; 0)	0.020
	Time from transplantation	0.1 (−0.6; 0.8)	0.802	−0.1 (−1; 0.8)	0.812
	Prior SARS-CoV-2 infection			24.5 (13.4; 35.7)	<0.0001
	Diabetes	−0.1 (−10; 9.9)	0.988	0.5 (−10.6; 11.5)	0.933
	Chronic renal failure	−11.2 (−21.3; −1.1)	0.031	−3.3 (−11.4; 4.8)	0.419
	Cancer	6.6 (−4.6; 17.7)	0.245	6.1 (−7.1; 19.2)	0.359
	Mycophenolate Mofetil Azathioprine	−2.6 (−11.5; 6.4)	0.567	5.1 (−1.8; 12)	0.141
	Triple immunosuppression	−9 (−18.5; 0.5)	0.064	−1.7 (−7.5; 4.1)	0.550
	Vaccine dose (post 3rd vs. 2nd dose)	21.2 (14.4; 27.9)	<0.0001	5.8 (1.3; 10.4)	0.013

β, regression coefficients; CI, Confidence interval; OLT, Orthotopic liver transplant; LUT, lung transplant.

^a^ Centred to the mean. Mean ages among the OLT recipients were 63.9 years in the anti-SARS-CoV-2 Ig analysis and 63.5 years in the INF-γ analysis. Mean ages among the LUT recipients were 45.1 years in the anti-SARS-CoV-2 Ig analysis and 45.3 years in the INF-γ analysis.

After the administration of the third dose, there were 60 BI recorded among LUT recipients and 68 among OLT recipients, observed over a total follow-up time of 31,933 days and 77,811 days, respectively. This corresponded to infection rates of 1.88 per 1,000 patient-days among LUT recipients and 0.87 per 1,000 patient-days among OLT recipients (IRR among LUT recipients: 2.15, 95% CI: 1.49–3.09). The majority of BI occurred in 2022 (124/128, 96.9%) and did not require hospitalization. Two OLT and seven LUT recipients were hospitalized due to severe COVID-19, whilst two LUT recipients and one OLT recipient died due to COVID-19. Four LUT and four OLT recipients died during the study period due to non-COVID-19 causes.

**FIGURE 2:**
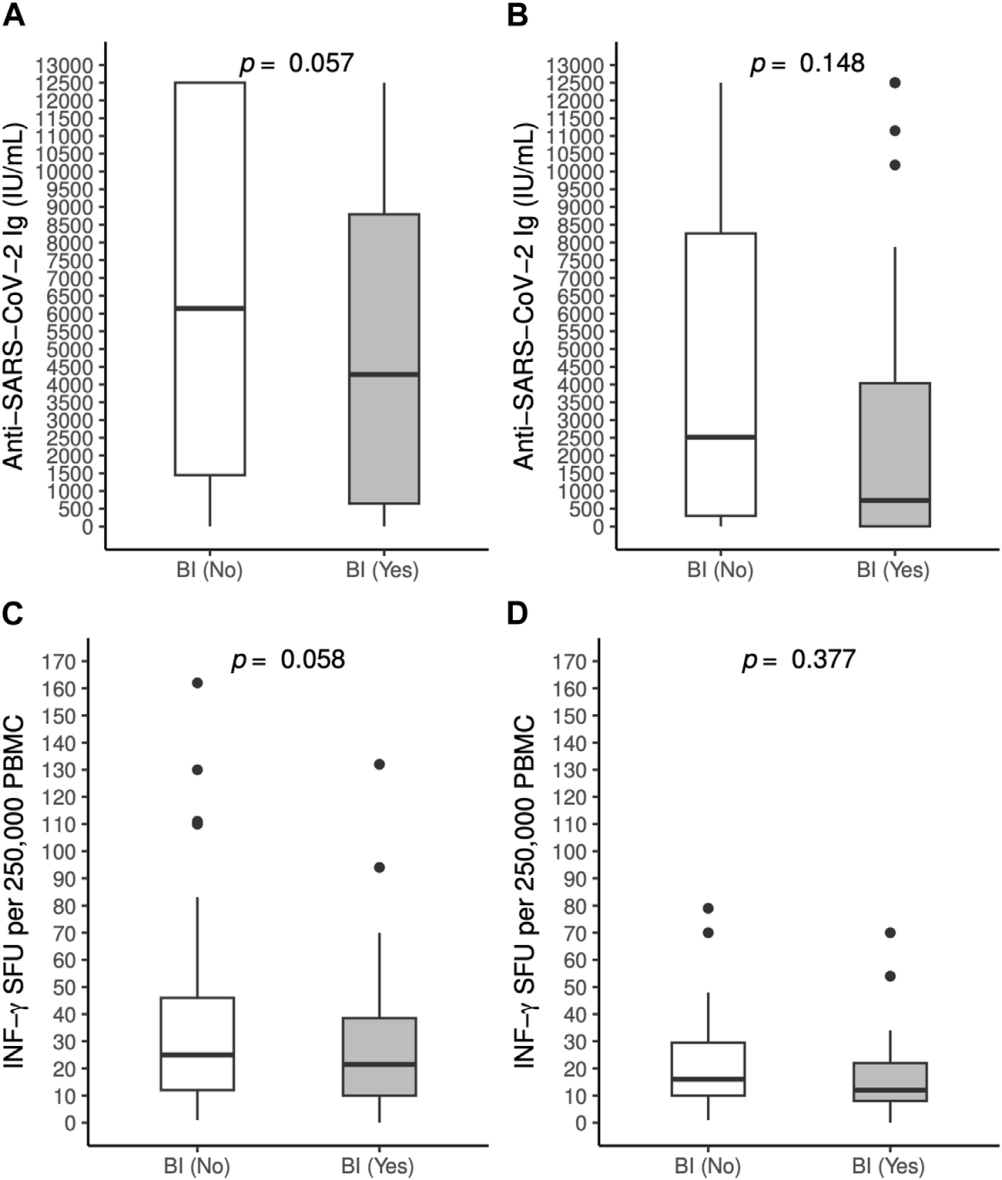
Antibody [**(A)**: OLT recipients, **(B)**: LUT recipients] and T-cell mediated responses [**(C)**: OLT recipients, **(D)**: LUT recipients] to BNT162b2 vaccine against SARS-CoV-2 measured 60 days post 3rd dose in patients who experienced a breakthrough infection and those who did not, by solid organ transplantation group. BI, Breakthrough infection; INF, Interferon, LTU: Lung transplant; OLT, Orthotopic transplant; PBMC: Peripheral blood mononuclear cells; SFU: Spot forming units.

Serum concentration of anti-SARS-CoV-2 antibodies and IFN-γ SFU after the third dose did not significantly differ among patients who reported a BI compared to those who did not (Figure 2).

No serious adverse event occurred; we report one case of transient leukopenia in a LUT patient after the administration of the second dose (spontaneous remission in the subsequent month).

## Discussion

In this large prospective study, we found that a two-dose course of the BNT162b2 vaccine elicited a positive immunogenicity in the majority (around 70%–80%) of OLT and LUT recipients included in our study. Humoral and T-cell mediated responses were higher among OLT recipients compared to LUT recipients. The administration of a third dose significantly enhanced both immune responses with positive response rates approaching 90% in LUT recipients and 95% in OLT recipients. No serious events were observed in both the transplanted cohorts.

Our findings may be somewhat unexpected. Long before the COVID-19 pandemic, several studies suggested that SOT recipients, given their immunocompromised state, tended to develop impaired immunogenicity to vaccination against viral pathogens and low overall response rates to other vaccines [9]. Following COVID-19 vaccination, lower serologic as well as T cell-mediated immune response were reported in SOT recipients when compared to the general population [5, 10].

In the ORCHESTRA cohort [11], OLT was associated with a significantly positive humoral response at 3 ± 1 months (79.1%), while lower rates of seroconversion were observed among LUT recipients (53.9%).

Limited data has been published regarding the role of T-cells in the protection against SARS-CoV-2 infection and this may be due to the complexity and cost of this technique if compared with serological analysis. In a study based on less than 40 transplanted patients, cellular immunity was more frequently found than humoral immunity (64.7%, vs. 35.3% for antibodies), suggesting that assessment of antibodies is probably insufficient to identify COVID-19-vaccine responders in SOT recipients [12]. Subsequent observations showed that this response could be enhanced after the administration of a booster dose. However, T cell response was still compromised when compared to that of healthy controls [13, 14].

Currently the two main methods used to conduct measurements of cellular immunity in vaccine studies are ELISpot and flow cytometry [15]. In our study, we used an INF-Y ELISpot assay, since its performance is maintained even in samples from patients with lymphopenia and immunosuppressed individuals [16]. ELISpot assay is already well known for its higher sensibility in cohorts of transplanted patients compared to other IGRAs [17, 18].

Our population is representative of general SOT recipients, as reflected in their demographic and clinical characteristics. However, the maintenance of a good graft function in the majority of our patients could partly explain the high rates of humoral and cellular responses in our population. Moreover, the better response in OLT recipients than LUT recipients may be linked also to the lower proportion of OLT recipients under a triple immunosuppressive treatment. These findings may reflect the pharmacodynamic effect of glucocorticoids regimen on INF-Y pathways secretion [19, 20]. Of note, in our cohort, chronic kidney disease was more frequent in LUT recipients than OLT recipients and low glomerular filtration rate was associated with lower antibody levels.

A daily dose of Mycophenolate Mofetil (MMF) > 1g/die could lead to a lower immunological response [12, 21]. In our study, we did not find a statistically significant association with the use of MMF, and this could be possibly explained by the low doses being used in our population.

To date, no definite conclusions can be drawn on the relationship between quantitative antibody measurement and protection from SARS-CoV-2 infection or COVID-19 disease.

Severe COVID-19 was uncommon in our cohort, with only a few patients requiring hospitalization (0.8% of OLT recipients and 5.8% of LUT recipients). The routine of both monoclonal antibodies and specific antiviral treatment early in the course of COVID-19 may have contributed to decrease the occurrence of severe and fatal cases [22, 23].

In the CONTRAST Study seroconversion occurred in the majority (78%) of 614 SOT patients, with an 18% incidence of BI. Levels of antibody response were associated with reduced risk of BI, while the burden of immunosuppressive drugs was not related with an increased risk of BI. OLT recipients were confirmed as being more likely to have a positive antibody response and a lower infection rate [24].

Data from the US Registry showed that SOT recipients have a higher risk of BI compared to the general population with the highest risk observed in LUT population (Hazard ratio, HR 2.11) and the lowest one in OLT (HR 1.39) recipients. The same study also showed a vaccine-related reduced mortality during BI for both general population and SOT recipients (HR 0.37 and 0.67, respectively) [25]. Our real-life data seems to confirm this trend as severe infections were uncommon in our cohort.

Overall, our findings highlight the importance of the third dose of COVID-19 vaccines as a booster. The benefits of booster doses are well established, both for COVID-19 [26, 27] but also for other vaccines, such as the inactivated polio vaccine [28].

### Strengths and Limitations

The large sample size is certainly the main strength of our study. We also provided unique data on the T-cell mediated immune response elicited by one of the most used COVID-19 vaccine in SOT recipients, using a highly performant method in this particular setting.

However, we acknowledge some limitations, including its single centre nature and the absence of healthy controls. While currently available scientific evidence supports the administration of a fourth and a fifth dose of vaccine against COVID-19 worldwide in patients with impaired immune system, we did not collect further data on serum level of antibodies and T-cell mediated immune response after these additional doses.

### Conclusion

The third dose of anti-COVID19 mRNA vaccine effectively enhanced both antibody and T-cell immune responses in SOT recipients. No significant risk factors related to lower responses were identified, and, more specifically, immunosuppressive therapy did not correlate to the grade of immune response elicited by the vaccination. Since it is well established that antibody levels tend to decrease linearly with time from vaccination, a strategy of repeated booster doses as indicated by official Institutions could be a valuable option to prevent the development of severe COVID-19 disease in the transplanted population.

## Data Availability

The raw data supporting the conclusions of this article will be made available by the authors, without undue reservation.

## Abbreviations

BI, Breakthrough Infections; BMI, Body Mass Index; CLAD, Chronic Lung Allograft Dysfunction; COPD, Chronic Obstructive Pulmonary Disease; COVID-19, SARS-CoV-2 disease; ELISpot, Enzyme Linked ImmunoSpot; IFN-γ, Interferon-gamma; ISHLT, International Society of Heart and Lung Transplantation; LUT, Lung Transplant; MMF, Mycophenolate Mofetil; OLT, Orthotopic Liver Transplant; PBMC, Peripheral Blood Mononuclear Cells; SAE, Serious Adverse Event; SFU, Spot Forming Units; SOT, Solid Organ Transplant.
