# Supplementary material for: COVID-19 Vaccine in Lung and Liver Transplant Recipients Exceeds Expectations: An Italian Real-Life Experience on Immunogenicity and Clinical Efficacy of BNT162b2 Vaccine
Source: Transpl Int. 2024 Jul 10;37:12729. doi: 10.3389/ti.2024.12729 (PMC11266016; doi:10.3389/ti.2024.12729)

Online SUPPLEMENT 1

LABORATORY PROCEDURES USED FOR THE QUANTIFICATION OF HUMORAL AND T CELL-MEDIATED IMMUNE RESPONSES

Humoral response

The detection of anti-SARS-CoV-2 antibodies titers was analyzed with two commercially available electrochemiluminescence immunoassay (ECLIA), Elecsys Anti-SARS-CoV-2 S and Elecsys Anti-SARS-CoV-2 (Roche Diagnostic, Switzerland) using serum samples according to the manufacturer's protocol.

Elecsys Anti-SARS-CoV-2 S was used to evaluate the SARS-CoV-2 humoral immune response elicited by BNT16262 vaccine by the quantitative detection of antibodies to SARS-CoV-2 spike protein RBD (anti-protein 1) total Ig at each timepoint (60 days after the second dose or after the third dose). The analytical measuring interval is 0.40-250 U/mL. The lower limit of quantification (LLOQ) was 0.4 U/mL; the upper limit of quantification (ULOQ) was 12,500 U/mL for 50-fold diluted samples. Values ≥ 0.8 U/mL were interpreted as positive per SARS-CoV2 anti-protein S1 total Ig (spike) antibodies.

Furthermore, Elecsys Anti-SARS-CoV-2 assay was used for the qualitative determination of antibodies against SARS-CoV-2 nucleocapsid protein (anti-N) total Ig at baseline and during the overall period of follow up to exclude asymptomatic infection in OLT and LUT recipients. A cutoff index (COI) ≥ 1.0 was interpreted as positive for SARS-CoV-2 anti-protein N total Ig (nucleocapsid) antibodies.

T cell-mediated immune response

SARS-CoV-2-specific T-cell mediated immunity was quantified using a commercially available T-SPOT Discovery SARS-CoV-2 kit (Oxford Immunotec Ltd, UK).

Peripheral blood mononuclear cells (PBMC) were isolated from the heparinized whole blood samples by density gradient centrifugation method. PBMC were suspended in culture medium and 250,000 PBMC /well were stimulated for 20 hours in 8-well strips precoated with an anti IFN-γ mouse monoclonal antibody with three SARS-CoV-2 specific antigen panels (SARS-CoV-2 spike (S), SARS-CoV-2 nucleocapsid (N) and SARS-CoV-2 membrane (M) specific antigen panel) and a fourth panel with a pool of SARS-CoV-2 epitopes with high degree of homology with endemic strains of coronavirus and phytohemagglutinin (PHA). IFN-γ ELISpot assay was performed according to manufacturer's instructions.

T cell-mediated immune response was quantified as IFN-γ spot forming units (SFU) per 250,000 PBMC using an automated ELISpot Reader System and ELISpot Software (Advanced Imaging Devices (AID), GmbH Germany) and was normalized by subtracting from IFN-γ SFU of antigen well the IFN-γ SFU of negative control well.

A positive response was defined by a result ≥ 8 IFN-γ SFU per 250,000 PBMC. This threshold represents the value corresponding to the mean plus two times the standard deviation of the IFN-γ SFU per 250,000 PBMC in the negative control.

Online SUPPLEMENT 2

The following flowchart shows the screening and enrollment procedure.

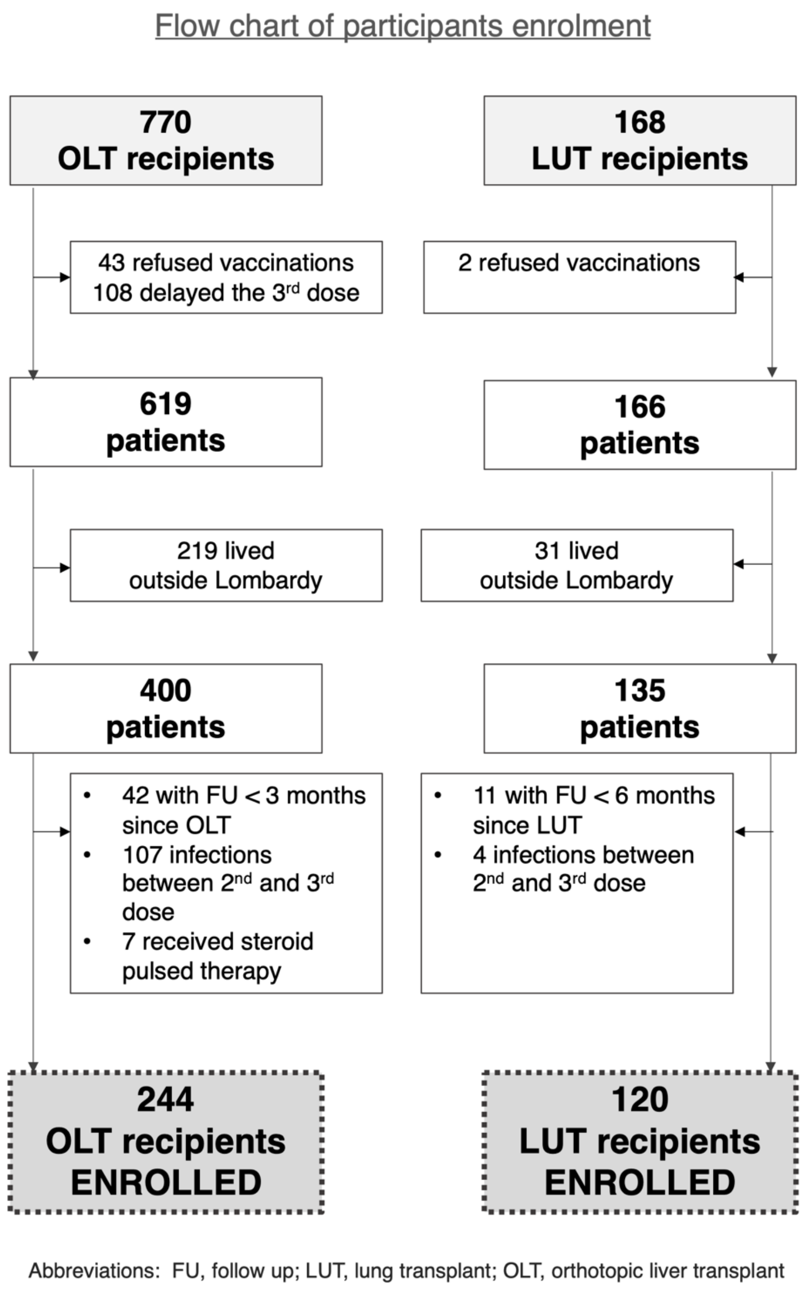

Supplement: Supplementary file 1 [file DataSheet1.pdf]
